# Effect of random feld disorder on topological superconductors

**DOI:** 10.1038/s41598-017-13158-w

**Published:** 2017-10-23

**Authors:** Tao Zhou

**Affiliations:** College of Science, Nanjing University of Aeronautics and Astronautics, Nanjing, 210016 China

## Abstract

We study the effect of random field disorder on two dimensional topological superconductors based on the Bogoliubov-de Gennes equations. A phase transition from the phase coherent state to the disordered state is identified numerically. The two phases can be characterized by two different correlation functions. In the phase coherent state, Majorana Fermion states form and may be influenced by the interaction between the vortex and the antivortex. The local density of states is calculated, which may be used to distinguish these two phases.

## Introduction

Topological superconductors have attracted great interest due to their nontrivial properties^1^. An important aspect is the excitation of the Majorana Fermions (MFs). It was indicated that the MFs exist at the system edges or vortex cores of *p* + *ip* topological superconductors^2^. Then it was verified theoretically that they should obey non-Abelian statistics^3^. Therefore, the realization of MFs is of significant interest and may have a potential application in the topological quantum computation^4^. In the past few years, a number of theoretical efforts have been made to probe the MFs in various topological superconducting system^5–14^. Experimentally, indications of MFs have also been reported by many groups^15–22^. While to date, a definite confirmation for the MFs and a direct demonstration of their non-Abelian statistics are still awaited.

The phase fluctuation is another important issue in the studies of unconventional superconductors. It may have a significant influence on both low-temperature properties and higher temperature normal state properties. Moreover, it is proposed to be related to the pseudogap phenomena in high-T_*c*_ superconductors^23–27^. Numerically, the phase fluctuations in high-T_*c*_ superconductors can be simulated phenomenologically by a classical XY model, and then some unusual properties can be soundly explained^24–27^. Within this framework, superconducting transitions should belong to the Kosterlitz-Thouless type phase transition^28^. For a Kosterlitz-Thouless type transition, the phase rigidity is broken by excitations of vortex-antivortex pairs. In topological superconductors, the vortex excitation is usually accompanied by the MFs excitations. Therefore, it may be rather interesting to study the phase fluctuation in topological superconductors, which has not been studied yet, mainly due to the fact that for materials with low superconducting transition temperatures, the thermal fluctuation effect in the superconducting state may be negligibly small. Moreover, a mean-field type calculation does not work well for the thermal fluctuation. It is therefore difficult to obtain the temperature dependence of the energy gaps and their phases strictly.

Besides the thermal fluctuation, phase fluctuations can also be induced by disorders. Actually, the disorder effect has been important in the studies of various condensed matter systems. Understanding the response to the disorder in topological phase may be crucial for interpreting some unusual physical properties and expanding the application of the real system. Interestingly, a well controlled random magnetic field has recently been realized experimentally^29,30^. Note that for superconducting systems the magnetic field should influent strongly on the phases of the order parameters. Thus, a random phase is expected to be introduced by a random field disorder. As a result, the flux in the order parameter phase may be induced, and then the MF states may be generated. It was also expected that the rigidity weakens and disappears in the presence of strong disorder, and thus a phase transition from the phase coherent state to the phase disordered state may occur. The disordered state in a superconducting system is an interesting issue and has attracted considerable interest in the past^31–33^. Therefore, it is rather important and essential to study the response and possible phase transitions in the presence of the random field.

In this paper, we study theoretically the random field effect (the average field keeps to be zero) on topological superconductors, where the disorder indeed introduces vortex pairs. The topological nature depends on the disorder strength *r*. For the case of weak disorder, the zero energy states appear to be protected by a minigap, corresponding to a pair of MFs. As the disorder strength increases, the vortex density increases. While different from the case of the uniform magnetic field effect, here the MF zero energy states are not bound to each vortex. Instead, a series of quasi-continuous low energy states appear. These low energy states are still protected by an minigap. Further increasing the disorder strength, the minigap disappears and the system enters into a disordered state.

## Results

In the presence of a magnetic field, the superconducting order parameter Δ_i_ is a complex quantity, with Δ_0i_ and *θ*_i_ being the amplitude and the phase, respectively (${{\rm{\Delta }}}_{{\rm{i}}}={{\rm{\Delta }}}_{0{\rm{i}}}{e}^{{\rm{i}}{\theta }_{{\rm{i}}}}$). Correspondingly, we can define the phase correlation function ${C}_{\theta }^{{\l}}$ and the amplitude correlation function ${C}_{{\rm{\Delta }}}^{{\l}}$, expressed as,

1 $$\begin{array}{c}{C}_{\theta }^{l}=|\langle exp[{\rm{i}}({\theta }_{{\rm{i}}}-{\theta }_{j})]\rangle |,\\ {C}_{{\rm{\Delta }}}^{l}=\langle {{\rm{\Delta }}}_{0{\rm{i}}}{{\rm{\Delta }}}_{0{\rm{j}}}\rangle ,\end{array}$$

where *l* is the distance between sites i and j ($l=|i-j|$). The angular brackets $\langle \cdot \rangle$ indicate the averages for the lattice sites i and j. Here the two functions depend on the disorder strength *r* and the distance *l*. Without disorder (*r* = 0), the superconducting order parameter is uniform with ${{\rm{\Delta }}}_{i}\equiv {{\rm{\Delta }}}_{0}$. We then have ${C}_{\theta }^{{\l}}\equiv 1$ and ${C}_{\Delta }^{l}\equiv {|{\rm{\Delta }}}_{0}{|}^{2}$. In the presence of disorder, the function ${C}_{\theta }^{{\l}}$ measures the phase coherence, which is important for achieving superconductivity of the system. The amplitude correlation function ${C}_{\Delta }^{l}$ is used to define the disordered state. When the superconductivity is broken by disorder, we expect that, in this state, ${C}_{\theta }^{l}$ approaches to zero and ${C}_{\Delta }^{l}$ approaches to a finite constant as $l\to \infty$.

**Figure 1:**
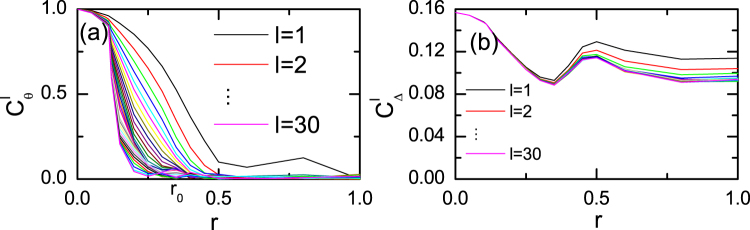
Gap phase correlation functions and amplitude correlation functions vs disorder strength.

The two correlation functions with different disorder strengths *r* and site distances *l* are displayed in Fig. 1. The phase correlation functions ${C}_{\theta }^{l}$ are plotted in Fig. 1(a). Generally, ${C}_{\theta }^{l}$ decreases as *r* and *l* increase. When the disorder strength is small, ${C}_{\theta }^{l}$ saturates to a finite value as *l* increases, indicating phase coherence and the existence of the long range order of the system. The phase rigidity weakens as the disorder strength increases and a critical strength value ${r}_{0}=0.35$ is revealed, at which the rigidity disappears completely. Then the system enters into a phase disordered state. The amplitude correlation functions ${C}_{{\rm{\Delta }}}^{l}$ are plotted in Fig. 1(b). As is seen, in the phase coherent state $(r < {r}_{0})$, ${C}_{{\rm{\Delta }}}^{l}$ decreases as *r* increases. Meanwhile, ${C}_{{\rm{\Delta }}}^{l}$ depends weakly on the site distance *l* when *r* is small. It reaches the local minimum at the critical point $r={r}_{0}$. While in the phase disordered state, relatively it depends weakly on the disorder strength. On the other hand, ${C}_{{\rm{\Delta }}}^{l}$ decreases as *l* increases. While, even for a fully disordered case (*r* = 1), ${C}_{{\rm{\Delta }}}^{l}$ will saturate to a finite value for $l\ge 5$. The above results indicate that the system enters into another phase disordered state as $r > {r}_{0}$, and ${C}_{{\rm{\Delta }}}^{0}$ may be used to describe this state.

**Figure 2:**
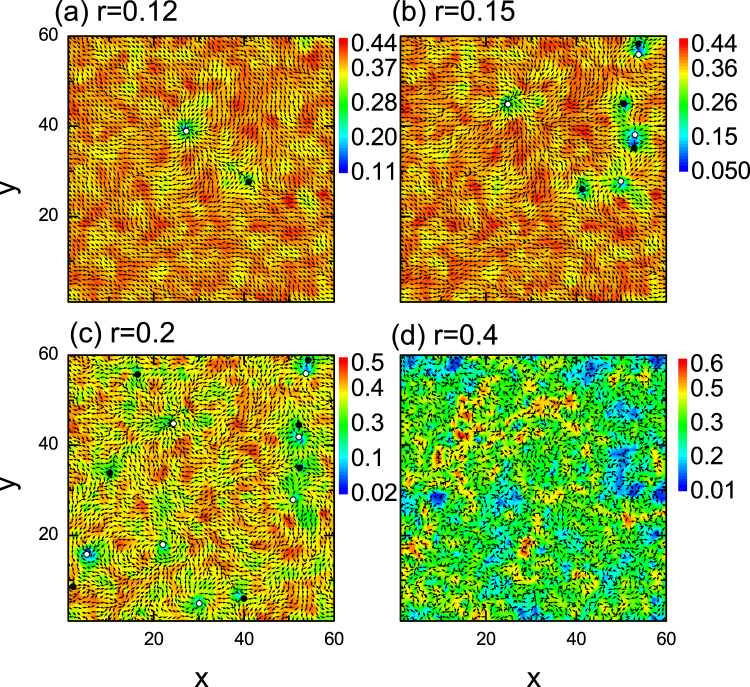
Amplitudes and phases of the superconducting order parameter with different disorder strengths. The vortex and antivortex are indicated by solid and open circles, respectively.

We now present the self-consistent results for the order parameters in order to obtain an intuitive understanding of the phase transition induced by the disorder. The amplitudes and phases (indicated by arrows) of the order parameters with different disorder strengths are presented in Fig. 2. We can see clearly that the vortices are excited as disorder is induced. Here two kinds of vortex, also named as vortex (winding along the clockwise) and antivortex (winding along the anticlockwise), can be obtained in Fig. 2. As is seen, for all of disorder strengths considered, the vortex and antivortex are excited in exactly equal proportions. They distribute randomly in space and no apparent order exists. For the phase coherent state, as shown in Fig. 2(a–c), the order parameter amplitude is suppressed significantly near the vortex. The number of the vortex/antivortex pairs increases monotonously as the disorder strength increases. Obviously, the phase rigidity will be weakened by the vortex. Thus, it is understandable that, for larger disorder strength, the phase rigidity disappears completely and the system enters into a phase disordered state. For this state, as is seen in Fig. 2(d), a number of vortices have been excited. However, significantly different from the case of the ordered state, that is, in the disordered state, the gap amplitudes are not suppressed at the vortex core.

**Figure 3:**
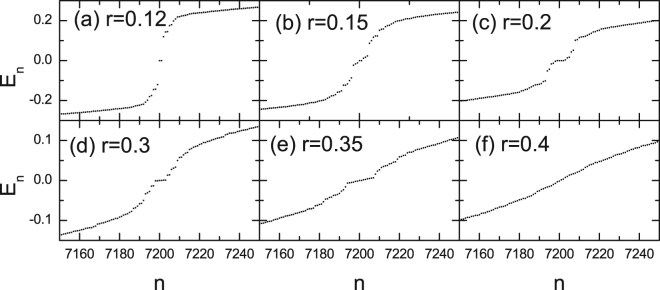
Numerical results of the eigenvalues with different disorder strengths.

Let us conduct a more systematic and accurate numerical study on the nature of the phase transition induced by the disorder. By diagonizing the BdG Hamiltonian, we can obtain 14400 eigenvalues. Due to the particle-hole symmetry, the eigenvalues always appear in pairs with energies +*E* and −*E*, respectively. The low energy eigenvalues from the BdG Hamiltonian are plotted in Fig. 3. For the case of weak disorder *r* = 0.12, as shown in Fig. 3(a), two zero-energy eigenvalues are revealed, protected by a minigap about 0.1. For this disorder strength, we have shown in Fig. 2(a) that there exists two vortices. Actually, the effect of a vortex in a topological superconductor has been previously studied intensively. Generally, each vortex binds a topological protected single zero-energy Majorana mode^34^. Thus the existence of the zero energy state is consistent with the numerical results for the order parameters shown in Fig. 2(a). As the disorder strength increases to 0.15 [Fig. 3(b)], there are eight low energy eigenvalues. These low energy states are also protected by an energy gap. This result in also consistent with the numerical result for the order parameter shown in Fig. 2(b), where eight vortices exist for *r* = 0.15. Rather interestingly, here only two zero energy eigenvalues exist, which indicates that the interaction between the vortex and antivortex may annihilate the MF zero states. Note that this is different from the case of the zero energy states introduced by the uniform field. As the disorder strength increases to *r* = 0.0 and *r* = 0.3$r=0.3$ [Fig. 3(c) and (d)], more low energy states are generated. Generally, the low energy states are protected by an gap and the number of the low energy states are consistent with that of the vortices. At the critical point (*r* = 0.35), where the order-disorder transition occurs, as is seen in Fig. 3(e), the minigap almost disappears and only a kink occurs at low energy. We also present the numerical result for the disordered state (*r* = 0.4), as is seen in Fig. 3(f), the eigenvalues are continuous and no any anomalous behavior exists at low energies.

**Figure 4:**
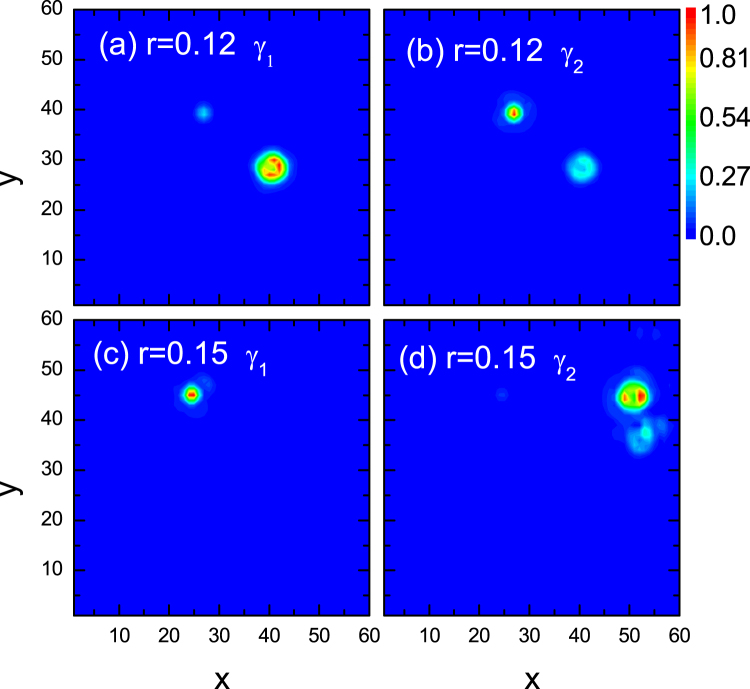
Spatial distributions of the two Majorana Fermions with disorder strengths *r* = 0.12 and *r* = 0.15, respectively.

As is known, the two zero energy eigenvalues in topological superconductors, with the operators expressed as *C* and *C* ^†^, are usually associated with two MFs. The operators of the two MFs are obtained by ${\gamma }_{1}=(C+{C}^{\dagger })$ and ${\gamma }_{2}=i({C}^{\dagger }-C)$. The distributions of the two MFs with the disorder strengths 0.12 and 0.15 are plotted in Fig. 4. As is seen, two locally separated MFs are identified numerically. For the case of *r* = 0.12, there exists a small overlap for the two MF states. This means that an effective interaction exists between the two MFs. The overlap seems interesting, and it may be influenced by the disorder strength. As the disorder strength increases to 0.15, the overlap disappears and the two MFs are completely separated. The existence of the MF states is important for identifying the topological nature of the ordered state.

## Discussion

**Figure 5:**
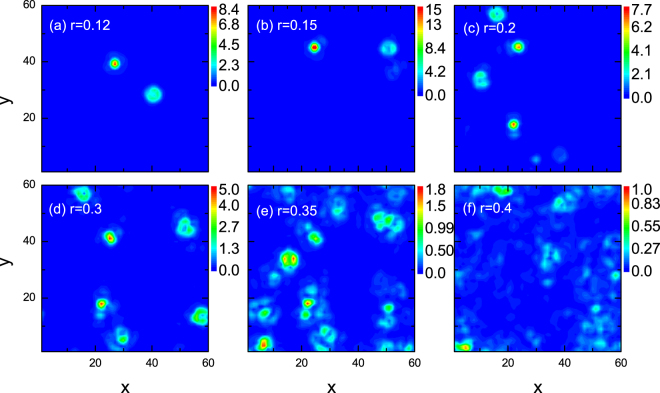
Intensity plots of zero energy LDOS spectra with different disorder strengths.

We have presented the numerical results and verified that the topological phase transition is induced by the random field. For weak disorder strength, MFs are excited and there exist low energy states protected by the minigap, indicating the topological nontrivial features. Moreover, the random field effect is of fundamental interest since the vortex-antivortex pairs are induced by the random field. Thus an effective K-T type phase transition may be simulated. One important issue is how to verify our above numerical calculations experimentally. Generally the existence of MFs can be detected via the presence of zero energy peaks in the LDOS spectra. The LDOS can be measured through scanning tunneling spectroscopy. We now investigate the LDOS to disclose a possible experimental observation of the low energy states and to clarify how to detect the differences between phase coherent state and phase disordered state experimentally. The intensity plots of the zero energy LDOS spectra with different disorder strengths are displayed in Fig. 5. As the disorder strength is small, the zero-energy LDOS is qualitatively the same with the spatial distribution of the two MFs. As the disorder strength increases, the maximum intensity decreases rapidly, indicating that the quasiparticle nature reduces. For the case of *r* = 0.4, no sharp zero energy peak exists, indicating that there are no zero energy states and MFs excitations. Based on these results, we propose that the zero energy LDOS can be used to differentiate the phase coherent state and the disordered state.

We would like to remark the significance of the present work. First, the topological phase transition in topological superconductors is of fundamental importance and has attracted broad interest previously. It may extend our understanding of several important physical concepts. Here our numerical calculations indicates that the order-disorder transition indeed occurs, and the topological non-trivial behaviours in the ordered state are identified through the existence of topological protected low energy states, and of the spatial separated MF states. Second, phase rigidity is an important aspect in the studies of superconductivity. Previously, phase rigidity in topological superconductors has not been studied yet. Random field disorder provides an effective way to study this issue. On the other hand, random field disorder can be realized artificially, and thus our calculation may be relevant to a real system. Third, random field disorder is different from other kinds of disorder. It introduces frustration, after which the system may enter a disordered state. Moreover, vortices form in presence of random field disorder. As a result, the MFs are introduced by the random field, which is rather interesting. The vortex bounded MFs in topological superconductors should obey non-Abelian statistics and have potential application in topological quantum computation. Thus the study of MFs is also of broad interest. In particular, as reported herein, one pair of MFs is bounded by one vortex and one antivortex. This situation is different from that in which MFs are induced by the uniform field. The interaction between the vortex and antivortex may annihilate the MFs, and then the zero energy bound state may shift to a finite energy state. Therefore, studying the random field may provide an effective method of understanding how the MFs are created and annihilated. This issue is of interest and merits further studies.

In summary, we have studied theoretically the effect of random field disorder on topological superconductors. An order-disorder transition is revealed numerically. The vortex/antivortex pairs are induced by the random field. In the ordered state, low energy states protected by a minigap exist. The MFs may be bounded by the vortex, which are identified numerically. However, here the MFs is different from those induced by the uniform field, i.e., the interaction between the vortex and antivortex may influence the MFs. Two MFs may be annihilated and zero bound states may shift to low finite energy states when the vortex pairs approach. In disordered state, the minigap closes and no zero energy bound state exists.

## Methods

We expect that our main results are robust with an effective model to describe the topological superconductors. The quantitative details of the model are not important. In the present work, we consider an effective model that describes the two-dimensional topological superconductors^6,9,14^, expressed as,

2 $$\begin{array}{c}H=-\sum _{{\rm{i}},\alpha }[{t}_{{\rm{i}},\alpha }{\psi }_{{\rm{i}}}^{\dagger }({\sigma }_{0}-i\lambda {\sigma }_{\alpha }){\psi }_{{\rm{i}}+\hat{\alpha }}+h\mathrm{.}c\mathrm{.]}\\ \quad \quad +\sum _{{\rm{i}}}{\psi }_{{\rm{i}}}^{\dagger }(h{\sigma }_{z}-\mu {\sigma }_{0}){\psi }_{{\rm{i}}}\\ \quad \quad +\sum _{{\rm{i}}}({{\rm{\Delta }}}_{{\rm{i}}}{\psi }_{{\rm{i}}}^{\dagger }i{\sigma }_{y}{\psi }_{{\rm{i}}}^{\dagger }+h\mathrm{.}c\mathrm{.),}\end{array}$$

with ${\psi }_{{\rm{i}}}={({\psi }_{{\rm{i}},\uparrow },{\psi }_{{\rm{i}},\downarrow })}^{T}$. $\hat{\alpha }=\hat{x}$ and $\hat{y}$ represent the base vector along the *x* and *y* directions. *h* is the Zeeman field and *λ* is the spin-orbital strength. ${\sigma }_{x,y,z}$ and *σ*_0_ are the Pauli matrix and identity matrix, respectively.

In the presence of random field, the hopping integral *t*_i,*α*_ is expressed as ${t}_{{\rm{i}},\alpha }={t}_{0}exp(i{A}_{{\rm{i}},\alpha })$. The random variables ${A}_{{\rm{i}},\alpha }$ are magnetic bond angles that are uniformly distributed within $[-r\pi ,r\pi ]$ with *r* being the disorder strength $\mathrm{(0}\le r\le \mathrm{1)}$.

The Hamiltonian Eq. (1) can be expressed as the 4*N* × 4*N* matrix (*N* is the number of the lattice sites), which can be diagonalized by solving the Bogoliubov-de Gennes (BdG) equations as,

3 $$\sum _{{\rm{j}}}(\begin{array}{cccc}{H}_{{\rm{i}}{\rm{j}}\uparrow \uparrow } & {H}_{{\rm{i}}{\rm{j}}\uparrow \downarrow } & {{\rm{\Delta }}}_{{\rm{j}}{\rm{j}}} & 0\\ {H}_{{\rm{i}}{\rm{j}}\downarrow \uparrow } & {H}_{{\rm{i}}{\rm{j}}\downarrow \downarrow } & 0 & -{{\rm{\Delta }}}_{{\rm{j}}{\rm{j}}}\\ {{\rm{\Delta }}}_{{\rm{j}}{\rm{j}}}^{\ast } & 0 & -{H}_{{\rm{i}}{\rm{j}}\downarrow \downarrow } & -{H}_{{\rm{i}}{\rm{j}}\downarrow \uparrow }^{\ast }\\ 0 & -{{\rm{\Delta }}}_{{\rm{j}}{\rm{j}}}^{\ast } & -{H}_{{\rm{i}}{\rm{j}}\uparrow \downarrow }^{\ast } & -{H}_{{\rm{i}}{\rm{j}}\uparrow \uparrow }\end{array})\begin{array}{c}{{\rm{\Psi }}}_{{\rm{j}}}^{n}\end{array}={E}_{n}\begin{array}{c}{{\rm{\Psi }}}_{{\rm{j}}}^{n}\end{array},$$

where ${H}_{{\rm{ij}}\sigma \sigma }$ and ${H}_{ij\sigma \bar{\sigma }}$ $(\sigma \ne \bar{\sigma })$ represent the hopping term and the spin-orbital coupling, respectively. ${E}_{n}$ $(n=\mathrm{1,}\,\mathrm{2,}\,\mathrm{3,}\,\cdots ,\,4N)$ are the eigenvalues of the Hamiltonian. ${{\rm{\Psi }}}_{n}={({u}_{{\rm{i}}\uparrow }^{n},{u}_{{\rm{i}}\downarrow }^{n},{v}_{{\rm{i}}\downarrow }^{n},{v}_{{\rm{i}}\uparrow }^{n})}^{T}$ (with i from 1 to *N*), are the corresponding column eigenvectors.

The superconducting pairing order parameters are determined self-consistently,

4 $${{\rm{\Delta }}}_{{\rm{i}}}=\frac{V}{2}\sum _{n}{u}_{{\rm{i}}\uparrow }^{n}{v}_{{\rm{i}}\downarrow }^{n\ast }\,\tanh (\frac{{E}_{n}}{2{K}_{B}T}),$$

with *V* being the pairing strength coming from the onsite attraction.

The site dependent electron density is calculated after diagonalizing the BdG Hamiltonian,

5 $${n}_{{\rm{i}}}=\sum _{n\sigma }|{u}_{{\rm{i}}\sigma }^{n}{|}^{2}f({E}_{n}\mathrm{).}$$

Here *f*(*x*) is the Fermi distribution function.

The local density of states (LDOS) can be calculated numerically as

6 $${\rho }_{{\rm{i}}}(\omega )=\sum _{n}[|{u}_{{\rm{i}}\uparrow }^{n}{|}^{2}\delta ({E}_{n}-\omega )+|{v}_{{\rm{i}}\downarrow }^{n}{|}^{2}\delta ({E}_{n}+\omega \mathrm{)].}$$

Here the delta function $\delta (E)$ is expressed as $\delta ={\rm{\Gamma }}/[\pi ({E}^{2}+{{\rm{\Gamma }}}^{2})$]. Γ is the quasiparticle damping with ${\rm{\Gamma }}=0.005$.

In the presented results, we consider *t*_0_ the energy unit. The numerical calculations are performed on a two-dimensional 60 × 60 lattice with the periodic boundary condition. The other parameters are set as *μ* = −4, *h* = 0.6, *λ* = 0.5, and the pairing potential *V* = 5. Note that when $\lambda \ne 0$, our starting Hamiltonian [Eq. (1)] is dual to a two-band *p*-wave superconductors through a unitary transformation^6^. Without the disorder, the superconducting pairing is uniform with ${{\rm{\Delta }}}_{{\rm{ii}}}\equiv {{\rm{\Delta }}}_{0}$. Then the system is in the topological non-trivial phase when the Fermi energy crosses the lower band and the upper band is completely empty. For the present chemical potential ($\mu =-4$), the topological feature are determined by the Zeeman field strength *h*. We have studied numerically and verified that ${h}_{c}=0.48$. The system is in the topological nontrivial phase when $h > {h}_{c}$. Therefore, our present model are an effective model to describe the topological superconductor.
